# Phenol-rich alternatives for *Rosa x damascena* Mill. Efficient phytochemical profiling using different extraction methods and colorimetric assays

**DOI:** 10.1038/s41598-021-03337-1

**Published:** 2021-12-13

**Authors:** Zuzanna Piotrowicz, Łukasz Tabisz, Marta Waligórska, Radosław Pankiewicz, Bogusława Łęska

**Affiliations:** Faculty of Chemistry, Adam Mickiewicz University in Poznań, Uniwersytetu Poznańskiego 8, 61-614 Poznań, Poland

**Keywords:** Analytical chemistry, Process chemistry, Environmental impact, Sustainability

## Abstract

Damask rose is a well-established, abundant source of phytochemicals, as well as economically important essential oil—however, its cultivation is demanding and costly. In this paper, extracts from four raw plant materials—*Salvia officinalis*, *Sambucus nigra*, *Matricaria chamomilla*, *Calendula officinalis*, known to be rich in phenolic compounds, but also far easier to cultivate—were directly compared to those obtained from *Rosa* × *damascena* Mill. By combining diverse extraction methodologies (in a Soxhlet apparatus, ultrawave-assisted and microwave-assisted, using supercritical CO_2_) and complementary in vitro assays (radical scavenging, iron reducing, Folin–Ciocalteau and Al^3+^ complexation), it was possible to conveniently approximate and compare the phytochemical portfolios of those diverse plants. By factoring in the crop yields of different species, economically important conclusions can be reached—with pot marigold (*C. officinalis*) seemingly the most viable substitute for damask rose as a source of phenolics. Fatty acid and microelement analyses were also performed, to further enrich the chemical profiles of plant extracts. The paper also aims to collate and redesign multiple colorimetric assays frequently used while studying plant extracts in vitro, but criticized for their lack of correlation to in vivo activity. We show that they remain a viable tool for direct comparison of extraction methodologies, while highlighting their shortcomings.

## Introduction

Damask rose (*Rosa* × *damascena* Mill., RD) is one of the best known and most popular sources of phytochemicals. Studies agree that rose extracts have very strong free radical scavenging properties (compared to other plants), which are correlated with the content of phenolic compounds^1^. To date, gallic acid, syryngic acid, quercetin, kaempferol and epicatechin have been identified in damask rose petals^2,3^. They also contain terpenes, glycosides and anthocyanins, carboxylic acids, vitamin C, tannins, and lipid compounds, including polyunsaturated fatty acids (PUFAs) and essential oils^4,5^. Phenolic compounds present in damask rose (e.g., epicatechin) have antioxidant and anticollagenase activity^6,7^. It is also reported that phenolic fraction extracted from damask rose shows a potent anti-hyperpigmentation effect^8^.

Damask rose is a cultivated plant. It is grown mainly on the Mediterranean coast, although most of the crops are located in the Rose Valley, Bulgaria. This species requires high air humidity, moderate temperatures and is fairly demanding in terms of soil quality and water^9^. Petals are usually hand-picked due to their extraordinary delicacy. All this makes the damask rose a demanding and difficult plant to cultivate, and therefore rose-derived products remain expensive. For this reason, the aim of this study was to find an alternative source of phenolics and other bioactive compounds among common species of herbaceous plants (in Europe and worldwide), which would be more easily available and cheaper to grow. While specific, standard-based methods are crucial in determining small differences in individual chemicals present in closely related plant species, and even between cultivars^10^, more general approach was deemed necessary to screen diverse raw materials for appreciable quantities of specific phenolic groups and other useful phytochemicals. Plants studied in this work were selected not only for their prevalence, but also for their common use as herbs, spices and flavoring agents, as well as in some nutraceuticals.

Elderberry (*Sambucus nigra,* SN) is a shrub commonly found throughout Europe, Central Asia, both Americas and North Africa. It grows wild, but it is also possible to find cultivars of this species^11^. Elderberry is one of the oldest plants used in medicine, as evidenced by Stone Age excavations, indicating early use of flowers and fruit for therapeutic purposes^12^. Flowers contain large amounts of flavonoids, such as kaempferol, astragaline, quercetin, quercetin-3-O-glucoside, rutin, isoquercetin and hyperoside^13^, and phenolic acids, i.e., ferulic, gallic, chlorogenic, syryngic and *p*-coumaric acids^14,15^. The other secondary metabolites are mainly triterpenes (e.g., α- and β-amirin, ursolic and oleanic acid), and sterols, such as campesterol, β-sitosterol and sigmasterol. Elderberry flowers contain pectins, tannins and minute amounts of essential oil, which includes ketones, alcohols, esters, oxides and terpenes^13^. Polyphenols obtained from this raw material show the ability to absorb UV radiation, reducing its penetration into deeper layers of the skin, thus preventing sunburn and DNA damage^16^.

Pot marigold (*Calendula officinalis,* CO) probably comes from the Mediterranean, where it is still found in natural habitats^17^. It is cultivated in many countries around the world, and sometimes also found in its feral form (ephemerophyte). Although it was used for therapeutic purposes already in antiquity, it is still used in modern herbal medicine, mainly in the case of burns, varicose veins, ulcers, jaundice and skin problems^18,19^. Among the most abundant compounds in pot marigold one can find phenolic compounds (including *p*-hydroxybenzoic, salicylic, caffeic and gallic acids, as well as acylated flavonoids, *O*-glycosides and methoxylated flavonoids) and saponins^20^. Marigold also contains carotenoids and triterpene alcohols, both in their free and esterified form, and PUFAs, such as calendic acid^17^, and proteins, amino acids, alkaloids, tannins, saturated hydrocarbons, high molecular weight polysaccharides, vitamin C, and minerals^20,21^. *C. officinalis* can also be a source of essential oil, of which approximately 25% is α-cadinol^22^.

*Calendula officinalis* flower extracts can be used in the treatment of inflammation and skin wounds due to its strong antimicrobial properties and antifungal activity^23,24^. Studies also suggest that pot marigold extracts can be beneficial for skin healing and procollagen synthesis^25^.

Chamomile (*Matricaria chamomilla,* MC) grows wild almost throughout the northern hemisphere (Europe, North America, and Asia), and even in Australia^26^; it also found as a cultivated plant. Chamomile was used in folk medicine since antiquity: to treat wounds, bruises, burns, migraines and also to alleviate nightmares, insomnia and as a mild sedative^27^. For therapeutic purposes, flower heads are collected. In chamomile flowers, over 120 chemical components have been identified as secondary metabolites, including terpenoids, flavonoids and other compounds with potential pharmacological activity^28^. These include: ferulic, caffeic, vanillic, protocatechuic, *p*-coumarinic, *o*-coumarinic and chlorogenic acids^29^. In turn, the following flavonoids are dominant: apigenin, quercetin, patuletin, luteolin and their glycosides^30^. Chamomile heads are also a source of essential oil, which contains a characteristic blue compound, chamazulene, which is synthetized from colorless sesquiterpene precursor, matricin^31,32^. Chamomile is one of the richest natural sources of apigenin, which influences a number of cellular processes, including cytokine production and the inflammatory response^33^.

Common sage (*Salvia officinalis,* SO) is a subshrub native to the Mediterranean, but it is currently grown all over Europe, including northern countries, such as Norway and Finland; sage plantations are also found in North America and Africa^34–36^. It is a valuable raw material in herbal medicine, foodstuffs and cosmetics. Sage was a symbol of health and longevity already in antiquity and is widely used as a medicinal plant, recommended for ailments related to pharyngitis, tonsillitis and gingivitis, as well as for other inflammatory conditions within the oral cavity^37–39^.

Various types of extracts rich in diterpenes (carnosol, carnosic acid, triterpenes (e.g. ursolic acid, oleanic acid), flavonoids (e.g. methyl derivatives of apigenin and luteolin) and phenolic acids are obtained from sage^40^. Common sage contains large amounts of rosmarinic acid (which, along with carnosol and carnosic acid, has the strongest antioxidant properties among all chemicals identified in plants of the genus *Salvia*)^41,42^. As for the other phenolic acids commonly found in herbaceous plants, such as gallic acid and ferulic acid, sage contains only small amounts^39^. Sage also contains vitamins—in particular vitamin C^43^—and essential oil^44^.

Rosmarinic acid protects against the harmful effects of UV radiation and ROS, shows antioxidant, anti-inflammatory, antiproliferative, antibacterial and even antiviral activity in vitro^45,46^. Carnosol is responsible for antioxidant and anti-inflammatory properties of sage extracts. In turn, carnosic acid has antimicrobial and antiobesity effects^42^.

This paper explores the similarities, differences and commercial viability of plants presented above as sources of phytochemical-rich extracts. For that purpose, four semi-qualitative, spectrophotometric assays, frequently used separately while studying plant extracts’ activities in vitro*,* were used in conjunction with four popular extraction methodologies: Soxhlet extraction (SOX), ultrasound- (UAE) and microwave-assisted extraction (MAE), as well as supercritical CO_2_ extraction (SFE). Despite the recent criticisms aimed at the so-called “in vitro antioxidant assays”—as they correlate poorly with in vivo activity—when combined together, they allowed for robust chemical profiling of selected plants. Redesigned in such a way, they can become a viable, efficient tool for determining the best extraction protocol for any given aim or phytochemical group—even when a diverse range of biomass samples is to be analyzed. Our research was further supplemented with microelement and fatty acid analyses of obtained extracts, to provide a more comprehensive outlook on studied species as sources of important chemicals for food, cosmetic and even pharmaceutical industries.

## Results and discussion

### Extraction methods and efficiency

**Figure 1:**
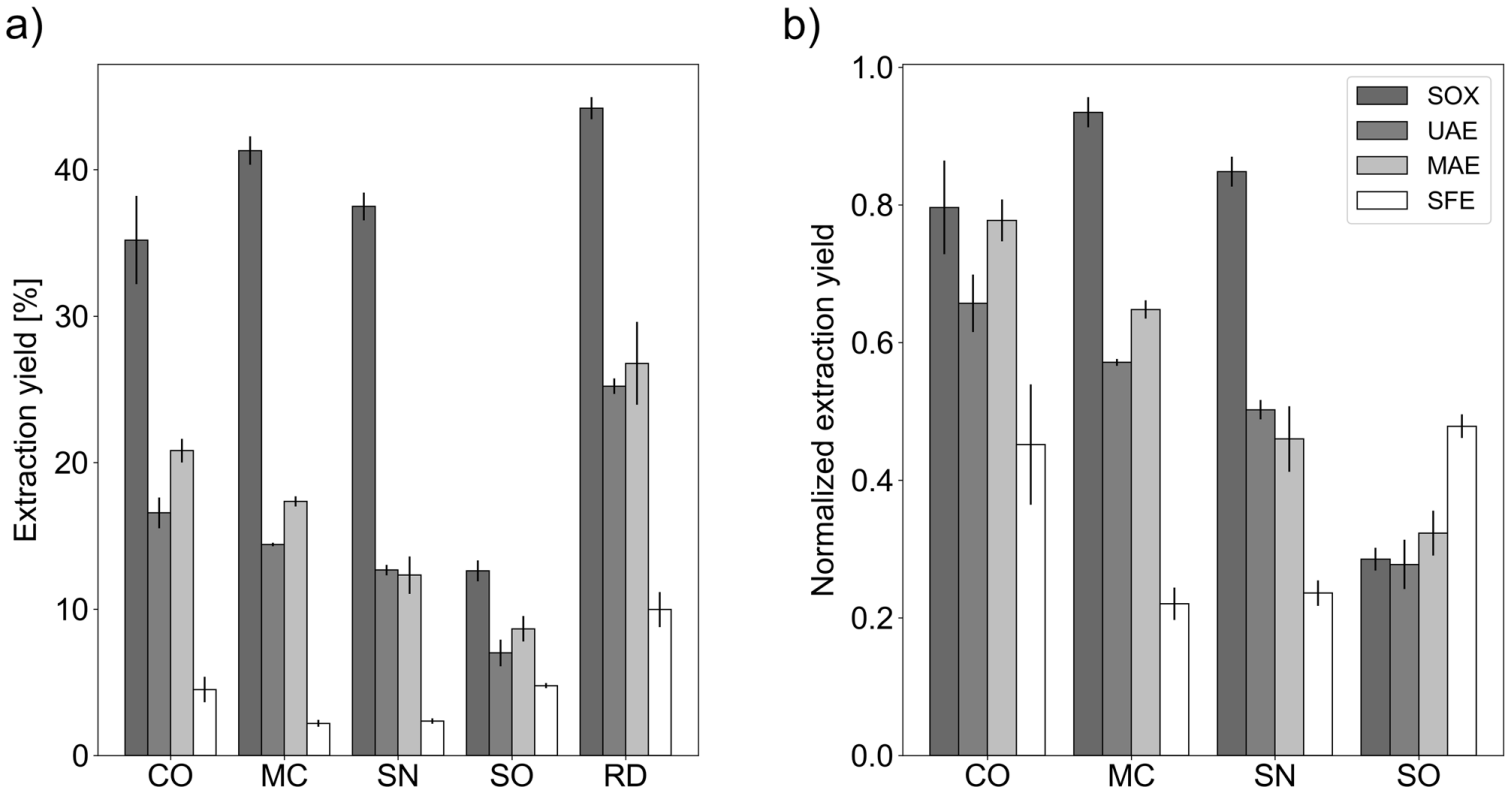
Yields of extracts prepared using different methodologies, given as (**a**) percent of dry plant material used and (**b**) fraction of extraction efficiency obtained for damask rose petals, when using the same extraction conditions. Error bars represent one standard deviation (extractions performed in triplicate).

Four commonly encountered plant biomass extraction methods were chosen for the study, and their yields compared (Fig. 1). Each was used in a manner suited to their particular strengths. The longest, energy-intensive extraction with a boiling solvent in a Soxhlet apparatus, lasting 24 h (SOX) can be treated as a point of reference for other methods, theoretically milder and/or faster. It was possible to isolate virtually all extractable compounds (soluble in ethanol) during the set timeframe: from 44% of sample mass in case of RD to 13% for SO.

Ultrawave-assisted (UAE) and microwave-assisted extractions (MAE) are both methods that should ensure better penetration of the solvent into the extracted material, in theory shortening the induction period and therefore the whole process^47^. With a quarter of SOX extraction time (i.e., 3 h), the yields were extremely similar for both methods, varying from 27% for RD-MAE to 7% with SO-UAE. As the drops in yields were universally much lower than drops in extraction times, it can be concluded that both UAE and MAE present viable, quicker alternatives to traditional, time-consuming extraction methods.

Supercritical fluid extraction (SFE) has been gaining more and more popularity, as it fits best the rising trend of green chemistry^48^. Especially CO_2_ as a solvent offers huge advantages, as it is cheap, non-toxic, and easy to recycle. However, the strictly nonpolar nature of CO_2_ limits the contents of SFE extracts, unless a co-solvent is used. Even then, the method’s flexibility is hindered, as shown by the low yields obtained: from 10% for RD to only 2% for MC (after 1 h). It can be therefore concluded that plant species selected for this study contain mostly polar or moderately polar extractable components, which can be successfully isolated using ethanol, but not supercritical CO_2_ (with the possible exception of SO, as discussed later).

The universally low extraction yields obtained for sage can be explained by the nature of the raw material itself—in this instance, biomass came from the entire plant, i.e. both the leaves and the stems. The latter especially contain high amounts of lignocellulose, which increases the weight of the raw material, while at the same time reducing the amount of extractable, active compounds. This poor performance is offsetted later, when the economical viability of SO crops is discussed.

### Redox-active compounds and phytochemical profiles of plant extracts

Apart from the quantitative results of extraction of different plant materials, when searching for phytochemical-rich biomass sources, the quality of extracts needs to be assessed as well. In the initial stage, where a wide variety of plants and extraction protocols are analyzed, the very time- and cost-intensive methods like chromatography can be unnecessary; the same is also true when a plant material is well-documented, but process optimization is underway. The so-called “in vitro antioxidant assays”, like the ABTS, FRAP, Folin–Ciocalteau (FC) and flavonol-Al^3+^ (FL-Al) methods, while popular^49^, have recently raised concerns—due to some authors claiming a correlation (now mostly disproven) between their results and in vivo activity^50^. Used together, however, they form an efficient tool for approximating the phytochemical profile of plant extracts. This battery of simple, fast, spectrophotometric determinations allowed us to draw broad conclusions concerning the relative amounts of different redox-active compounds—such as polyphenols, vitamins, and carotenoids.

**Figure 2:**
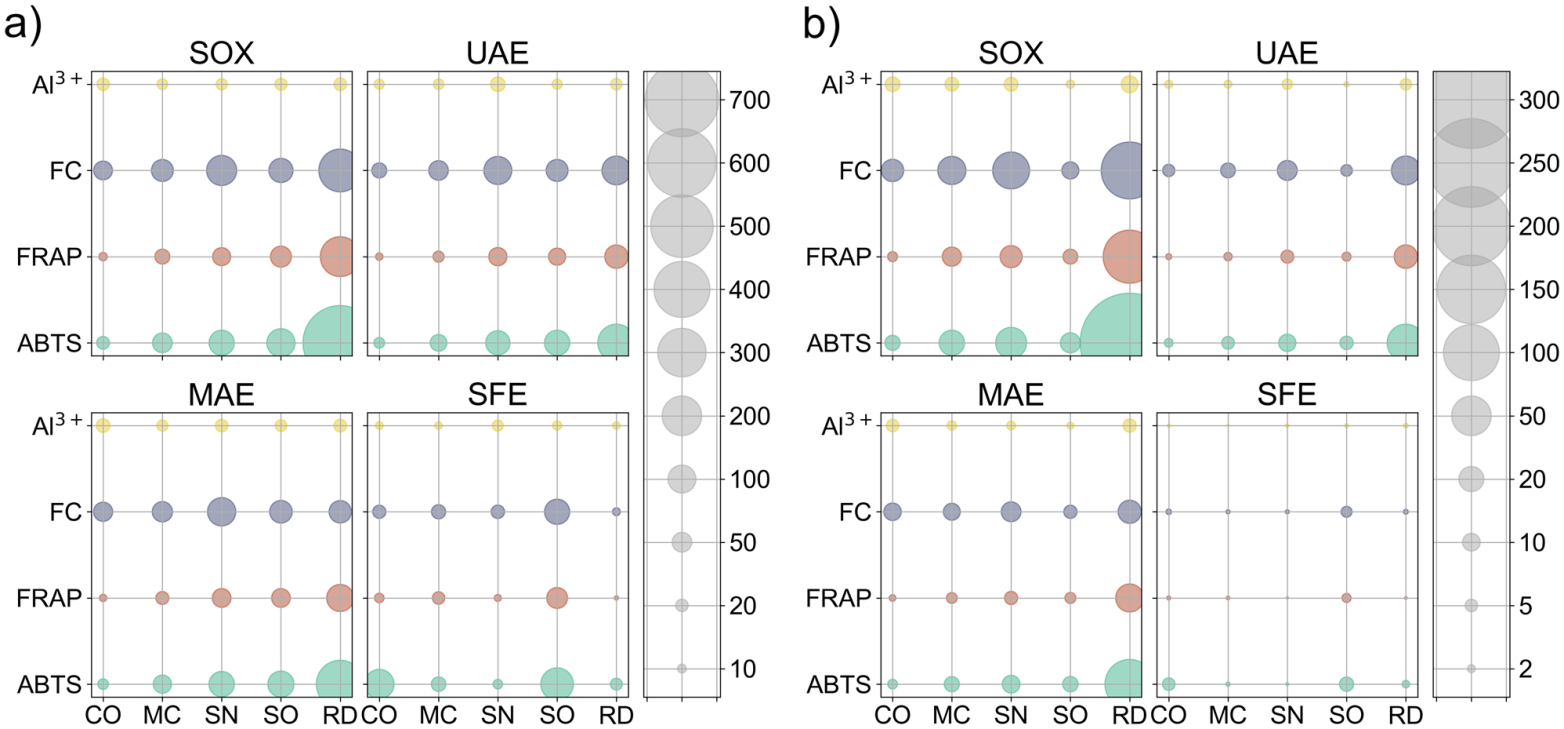
Comparison of activities of different extracts in all colorimetric assays, shown as milligram-equivalents of appropriate standard per gram of (**a**) pure extract or (**b**) raw plant material (see “Materials and methods” and Supplementary material for details). Error bars are omitted for clarity. All results (triplicate for each extract) were within ± 5% of each other.

**Figure 3:**
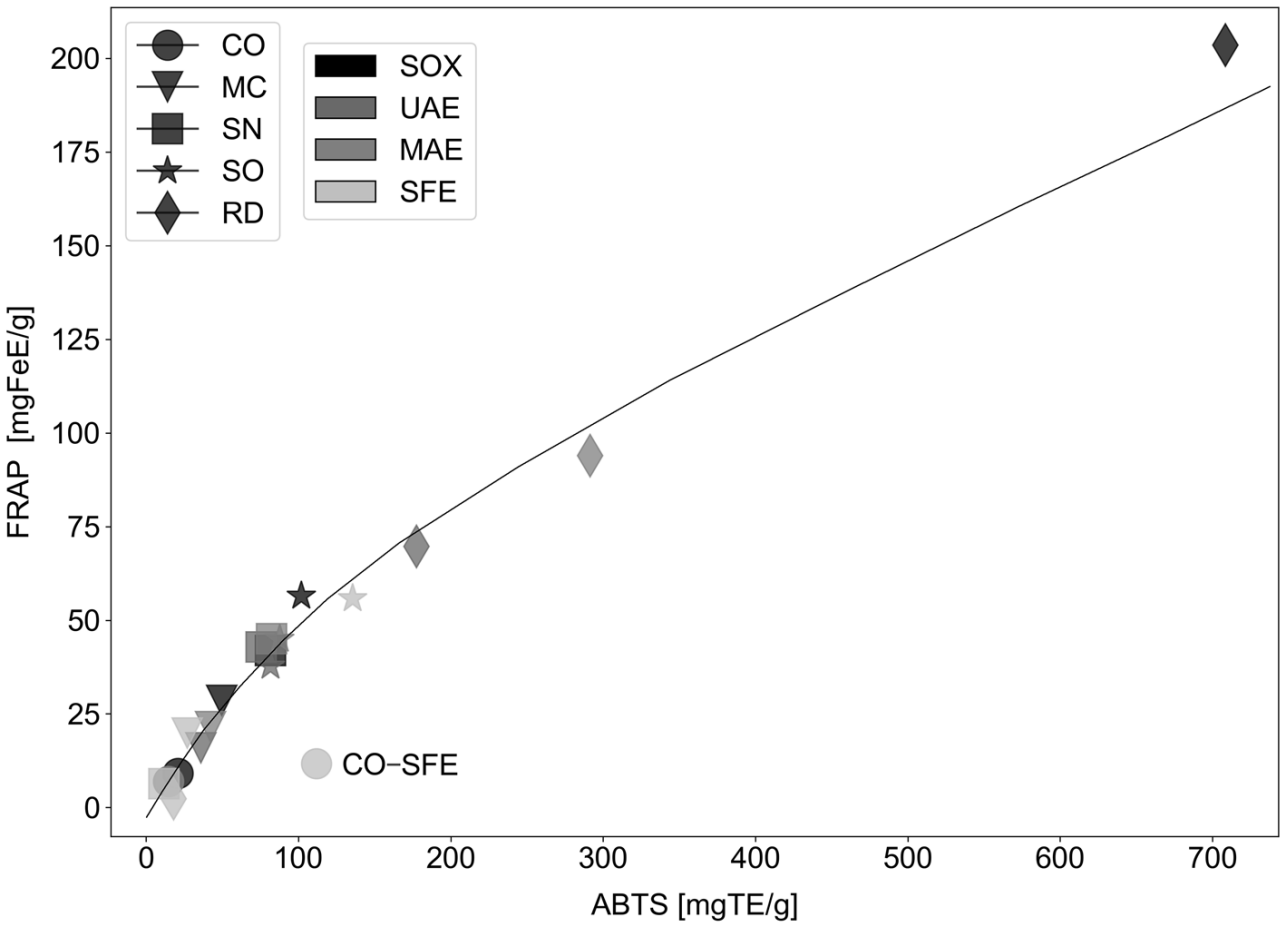
Correlation between FRAP and ABTS assays, showing a strong, but evidently non-linear interdependence. Additionally, FRAP assay appears to be far less sensitive to retinoid-type compounds, as evidenced by vastly out-of-trend position held by lipid-rich SFE extract obtained from *Calendula officinalis* (prepared using supercritical CO_2_).

The assays increase in selectivity in the order they were mentioned, which is fairly in line with numerically decreasing results that were obtained for most samples (Fig. 2). While ABTS and FRAP methods are commonly used interchangeably—in theory being both able to measure the ability of a compound to scavenge free radicals in vitro by any chemical means—their mechanisms differ, with the second one’s based on the reduction of Fe^3+^ ions. Determinations performed for the purposes of this study reveal some important deviations, especially when comparing results for *C. officinalis* extracts obtained by SOX and SFE methods (compare Figs. 2 and 3). In the latter case, the measured FRAP reducing activity is negligible, which contradicts the results from ABTS method. This leads to the conclusion that FRAP method is far less sensitive to the presence of retinoids, which are present in high concentration in CO, and can be expected to be especially abundant in CO-SFE. In the case of sage extracts, no significant differences were observed between those two extracts, which means that the non-polar radical scavenging compounds present in SO do not belong to the group of retinoids.

The details and rationale behind phytochemical profiling of plant materials and their extracts is outlined below, separately for each of the species.

**Figure 4:**
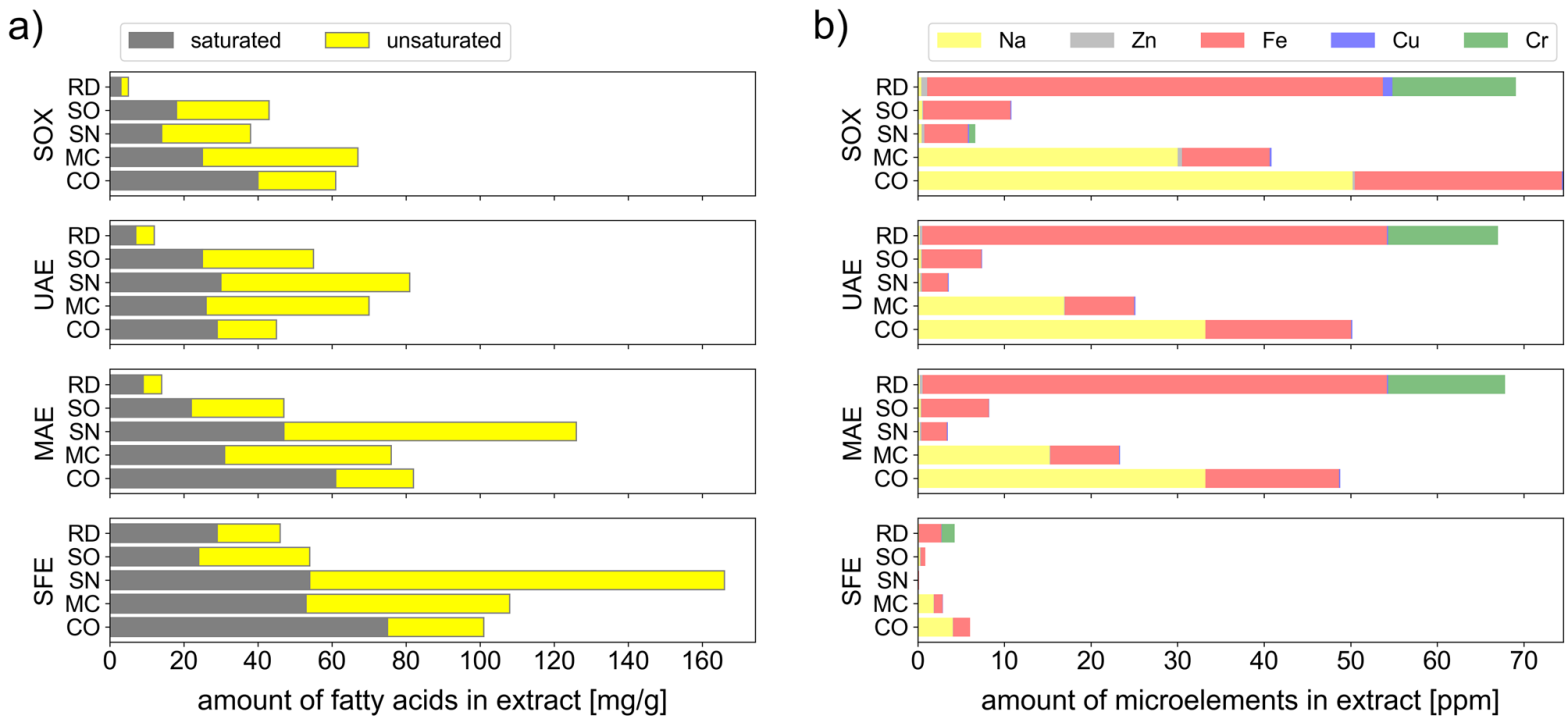
Additional information concerning the chemical composition of plant extracts obtained using different methods. (**a**) Amount of saturated and unsaturated fatty acids. (**b**) Amount of microelements. Error bars are omitted for clarity. All results (triplicate for each extract) were within ± 5% of each other. Precise, tabularized data are available in the Supplementary material.

The damask rose extracts, as expected, contained the highest concentration of redox-active compounds (results obtained from the most general ABTS and FRAP assays) in extracts obtained by all methods, except for SFE (Fig. 2). This suggests that damask rose contains the highest amount of polar or moderately polar in vitro antioxidants (which can be extracted using ethanol) and at the same time only relatively few non-polar compounds capable of radical scavenging, such as vitamin E and retinoids. The determination of phenol content using the FC method shows that they indeed comprise a very large part of extractable phytochemicals contained in the damask rose (rose extracts again exhibited the highest activities, among all of the examined extracts), which is consistent with the literature^2^. However, this material contains relatively few flavonols—or they exist in highly saccharified forms, which show lower activity in FL-Al assay (they are also more polar, yet still visible using the ABTS method). While the yield of RD-SFE was 2–5 times higher when compared to other SFE extracts, in combination with its assay activities and fatty acid content both being low (Fig. 4a), this indicates a substantial essential oil content (which is, likewise, well-documented in the literature)^4^.

Elderberry flowers contain high amounts of phenolic compounds (the activity of extracts in FC assay is second only to damask rose in most cases, and even highest in the case of MAE extracts—see Fig. 2), of which flavonoids are present in moderate amount (which can be concluded after comparing the results of FL-Al and FC assays)—which also stays in line with expectations^13^. These flavonoids are easily extracted, however, as can be seen by the results obtained for SN-UAE and SN-SFE. The low total yield of the SN-SFE, together with its high fatty acid and flavonoid content, leaves little room for essential oil, which is also supported by the literature^13^. Apart from that, data suggest a similar phenolic profile to that observed for damask rose; while elderberry extracts contain less total phenolics, they remain in strong second position overall.

On the other hand, the assay results and the efficiency of *C. officinalis* extraction suggest that the content of retinoids in this plant material is appreciably high. Comparable in vitro radical scavenging activities of CO-SOX and CO-SFE indicate that a significant amount of redox-active compounds contained in calendula are non-polar. Comparing this knowledge with the results of FRAP determinations, it can be safely stated that these compounds are mostly retinoids and similar polyunsaturated derivatives^51^. Attention should also be paid to the results obtained for CO from ABTS test. While ethanol extracts from pot marigold do not possess exceptional scavenging activity, CO-SFE actually shows similar activity to CO-SOX. It can be therefore concluded that most of the redox-active compounds found in this plant are indeed low-polarity compounds. Apart from them, calendic acid present in pot marigold (a fatty acid that exhibits antioxidant properties)^17^ also adds to the high activity of CO-SFE. Furthermore, on the basis of high yield of SFE extraction and measured fatty acid content, a large fraction of lipid compounds in calendula is also evident. In the case of polar in vitro antioxidants, there are proportionally few of these: phenolic compounds with a significant proportion of flavonoids comprise the majority of this group. Attention should be also paid to the results of the determination of the content of flavonoids by FL-Al method. When considered together with the results obtained using the Folin–Ciocalteau’s method, CO can be viewed as a particularly rich source of flavonols. All these observations are consistent with literature data^21^.

Among all of the studied plants, only MC-SOX was obtained with a yield approaching that of RD-SOX (Fig. 1b). The former showed lower activities in ABTS and FRAP assays (although higher than *C. officinalis* and *S. officinalis*) with phenolics constituting a significant amount of extractables—which can be concluded on the basis of the results of FC assay, and which is again consistent with literature^52^. Flavonols constitute a fairly small group among phenolic compounds in chamomile, and the literature data show that apigenin (which performs poorly in FL-Al assay) is the most abundant flavonoid in chamomile^30^. Based on the low yield of MC-SFE and the results of colorimetric assay activities shown by it, it can be concluded that the content of non-polar redox-active compounds in chamomile is fairly low. The main components of SFE extracts are fatty acids (Fig. 4a) and essential oil, of which one particularly interesting constituent, chamazulene, may be responsible for some activity shown in the ABTS method.

Ethanol extracts from sage were obtained with lowest yields among all of the tested raw materials, while SO-SFE extract was obtained with the second highest, after RD-SFE. The results of ABTS and FRAP determinations for SO-SOX were one of the lowest, while in the case of SO-SFE they were actually the highest. No significant differences were observed between results of these two assays, which means that the non-polar radical scavenging compounds present in SO do not belong to the group of retinoids. In this case, the results of the FC assay suggest that phenolic compounds constituted a large part of the phytochemicals, and were actually dominant in the SO-SFE. All of these data (as well as the results of fatty acid determinations) suggest that sage contains a lot of non-polar, mostly phenolic redox-active compounds. This is again strongly validated by the existing literature, which indicates a very high content of carnosol, as well as carnolic and rosmarinic acids in sage extracts^41^. These compounds can occur in the plant as aglycones, which makes them easily extractable with a non-polar solvent, such as supercritical CO_2_. Additionally, based on the results of complexometric method with Al^3+^, it can be safely assumed that sage has a relatively high content of flavonoids (flavonols), which represent a large proportion of the above-mentioned phenolic compounds—despite their lower overall SO-SOX concentration.

### Microelements in selected herbaceous plant extracts

In order to flesh out the chemical profile of plant extracts, the concentration of five elements (sodium, zinc, iron, copper, and chromium) was also determined (Fig. 4b). It should be noted that, while low in concentration, those metal ions could only appear in ethanolic extracts as organic complexes and other lipophilic conjugates, which are known for their high transdermal mobility and bioavailability^53^.

Calendula and chamomile extracts turned out to be the richest in sodium (~ 0.05 and ~ 0.03 mg/g, respectively). Sodium is essential for maintaining the membrane potential and the volume of body cells (Na^+^/K^+^-ATPase, so-called sodium–potassium pump), stimulating the nerves and muscles, and the correct osmotic pressure of body fluids^54^.

Damask rose, on the other hand, turned out to be a good source of iron (> 0.05 mg/g of extract). Calendula extracts also contained significant amounts of iron (~ 0.024 mg/g), and slightly smaller amounts of this element were present in sage and chamomile extracts (~ 0.01 mg/g). The iron-containing heme acts as a cofactor of hemoglobin and myoglobin proteins, both of which play the roles of oxygen carriers in the body^55^.

The remaining microelements were present in the tested plants in negligible amounts. The exact results of the determinations can be found in the Supplementary material.

### Cultivating selected plants as sources of phenolic compounds: economic aspect

**Figure 5:**
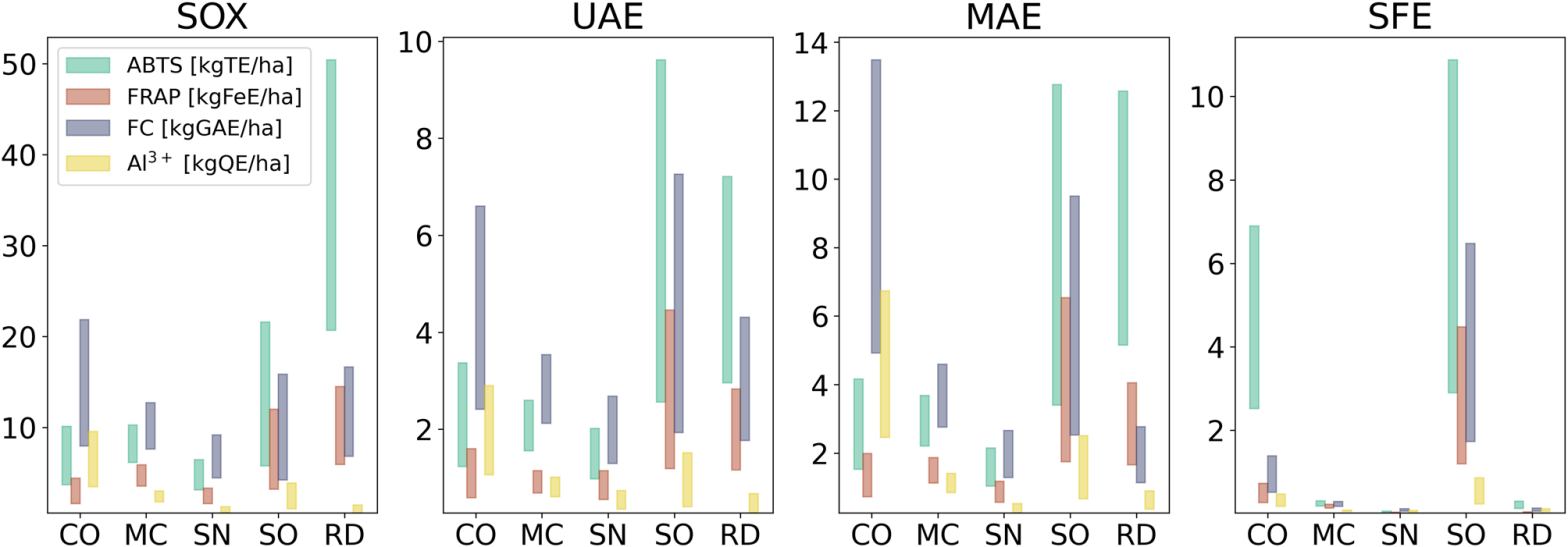
Potential yields of bioactive compounds, given as kilogram-equivalents of appropriate standard (see “Materials and methods” for details), per hectare of a given crop. The vertical bars denote the range of possible returns, as calculated using literature-reported crop yields of the five tested plants.

Based on available literature data^28,31,56–64^, an attempt was made to convert the reported crop yields of selected plants into amount of different phytochemicals/in vitro activities that could be obtained per hectare. The authors want to stress that the values given (Fig. 5) ​​should be treated as rough approximations, due to large differences in the reported yields of crops from different parts of the world. Nevertheless, some important and interesting conclusions can be drawn from the data.

Taking into account the total content of all in vitro radical scavengers, the greatest amount can be obtained from one hectare of damask rose cultivation. Surprisingly, however, the second place is taken by sage (25–40% of total scavenging activity supplied by RD-SOX obtained from the same cultivation area). It is also worth noting that in terms of producing SFE extracts, *S. officinalis* crops offer yields up to 60 times higher than those obtainable from other studied plants.

When considering selected plant materials as potential sources of phenolic compounds alone (FC assay), *C. officinalis* is the most productive crop; the same is the case when only flavonoids/flavonols are taken into account. In terms of obtaining these compounds, the cultivation of chamomile and sage may also turn out to be a worthwhile investment (flavonoids in pot marigold extracts amount to about 30% of those from calendula, when calculated per hectare). Also worth mentioning is the high amount of fatty acids in the inflorescences and seeds of calendula and chamomile^17^, when calculated per hectare. Although elderberry extracts showed high in vitro activities (second only to RD), their procurement from crops may not be as profitable. It is worth mentioning, however, that elderberry bushes are used to obtain both flowers (in spring) and fruit (in autumn). Although according to literature sources the latter have a lower content of phenolic compounds, they are a valuable source of vitamin C and anthocyanins^14,65^. SN is also undemanding and easy to grow, and flowers can also be obtained from plants growing commonly in the wild, even on an industrial scale.

## Conclusion

Herbaceous plants are a rich and varied source of phytochemicals, including phenolics and other redox-active compounds, which are in high demand in both food, nutraceutical and cosmetic industries. To balance industrial, economical and ecological needs, new plant sources must be analyzed for their potential to supply those phytochemicals, and the processing of well-known species’ biomass has to be optimized. For this purpose, a battery of well-known, spectrophotometric, semi-quantitative assays was compiled and redesigned as an efficient tool for screening and profiling of plant materials and their extracts. Combined with yields obtained with four extraction methodologies, as well as data from plantation studies, a new approach for comparing diverse crops (and their optimal processing methods) was established.

Unequivocally, the highest extraction yields and in vitro assay results were obtained for damask rose extracts. However, if we take into account the cultivation of tested plants in terms of phenolic compounds obtained, pot marigold crops actually become the most valuable. This species additionally contains significant amounts of important fatty acids and retinoids. Slightly less efficient would be the cultivation of chamomile (which is, however, a valuable source of apigenin) and sage (extremely rich in rosmarinic acid)—the latter being the most economical option for production of “green” supercritical CO_2_ extracts. In fact, due to the technological advances and incentives for removal of atmospheric carbon dioxide, this type of extraction is on track to become the most scalable and efficient in the near future. Of the five studied plants, the least productive, in terms of kilograms of phenolic compounds obtained per hectare, seems to be the cultivation of elderberry—although extracts obtained from this raw material showed the second highest (after the damask rose) activity in all of the performed assays. It is worth remembering, however, that crops from elderberry bushes can be obtained twice a year—in spring (flowers) and in autumn (fruit); this plant is also very easy to grow and common in the wild.

In summary, while rose remains the richest source of phenolic compounds and redox-active molecules, taking into account the efficiency and economic aspects of its cultivation, there are indeed less demanding, cheaper to grow, more accessible crops available as viable replacements. *Salvia officinalis*, *Sambucus nigra*, *Matricaria chamomilla* and *Calendula officinalis* can all be seen as valuable sources of bioactive extracts for food and cosmetic industries, and in some aspects—or when obtained under specific conditions—can even outshine the “gold standard” set by the damask rose. Despite the mounting criticism of “in vitro antioxidant assays”, they remain a valuable tool for discerning phytochemical profiles and optimization of biomass processing. Instead of discarding them, their shortcomings should be better studied, and methods themselves further developed—so their place in plant sciences can be fully realized, while not being overstated.

## Materials and methods

### Materials and reagents

#### Plant material

Five types of plant material were studied: dried damask rose petals (RD), dried chamomile flower heads (MC), dried calendula flowers (CO), dried elderberry flowers (SN) and dried and cut whole sage herb (SO). All raw materials were purchased from local qualified suppliers (Poland), with the exception of damask rose (imported from Rose Valley, Bulgaria). The study complies with local and national guidelines.

#### Reagents

Ethanol was used as the extraction solvent (96%, special purity, Avantor). Other solvents were of analytical or higher grade (Merck). In addition, the following reagents were used in colorimetric assays: Folin–Ciocalteau's reagent (analytical grade; Merck), sodium carbonate (> 99%; Avantor), potassium persulfate (> 99%; Merck), 2,2'-azino-bis(3-ethylbenzothiazoline-6-sulfonic acid) diammonium salt (ABTS; > 98%; Merck), 2,4,6-tris(2-pyridyl)-*s*-triazine (> 98%; Merck), Al(NO_3_)_3_ · 9H_2_O (> 99.9%; Merck) and as reference standards: gallic acid (> 99%; Merck), quercetin (≥ 95% by HPLC; Merck) and 6-hydroxy-2,5,7,8-tetramethylchroman-2-carboxylic acid ("Trolox"; > 97%; Merck). For the determination of fatty acids and elemental analysis, the following reagents were used: *t*-butyl methyl ether (> 99.8%), trimethylsulfonium hydroxide (0.25 M in methanol), methyl undecanoate (99%), Certified Reference Material Fatty Acid Methyl Ester (FAME) Standard, hydrochloric acid (37%), nitric acid (70%), and element standards (potassium, calcium, magnesium, sodium, chromium, iron, zinc, copper, silver, cobalt). All reagents were purchased from Merck.

### Extraction methodology

#### Soxhlet extraction (SOX)

About 20 g (in case of sage and elderberry) or about 10 g (in case of calendula, chamomile and damask rose) of non-comminuted, dry plant material were used in the extraction. The process was carried out for 24 h in a cellulose thimble, in a 250 mL apparatus using 300 mL of ethanol. Extracts were filtered hot and evaporated to constant weight under reduced pressure.

#### Ultrasound-assisted extraction (UAE)

To perform the ultrasound-assisted extraction, 20 g of dried sage and elderberry flowers, and 10 g of calendula, chamomile and damask rose flowers were weighed. The plant material was transferred to separate round bottom flasks and covered with 300 mL of ethanol. Extraction was performed in an ultrasonic bath (Sonic 10, Polsonic, Poland) at 40–45 °C for 3 h. The alcoholic extracts were filtered and evaporated to constant weight under reduced pressure.

#### Microwave-assisted solvent extraction (MAE)

In order to perform the extraction, about 2 g of dried sage and elderberry, and about 1 g of dried damask rose, chamomile and calendula were weighed. The samples were transferred into Teflon bombs, to which 30 mL of ethanol were added. The extraction procedure was carried out in a microwave oven (MARSXpress, CEM, USA) at a temperature of 40 °C (with a power of 400 W) for 180 min. The extracts were filtered and evaporated to constant weight.

#### Supercritical carbon dioxide extraction (SFE)

To perform the extraction, about 4 g of dried elderberry, 1.2 g of dried calendula, 1.3 g of dried damask rose, 2 g of dried chamomile, and 3.2 g of dried sage were weighed. The weighed raw materials were transferred to extraction thimbles, which were placed in a supercritical fluid extraction apparatus (MV-10 ASFE, Waters, USA). The starting parameters for the process were 50 °C and 200 bar. Extraction was carried out under these conditions for 60 min. The flows of CO_2_ and co-solvent (ethanol) were 8 mL/min and 0.8 mL/min, respectively. The obtained extracts were evaporated to constant weight under reduced pressure.

### Semi-quantitative colorimetric assays

In all cases, known and commonly used methods were modified in order to better adapt them to the specificity of research carried out, equipment, as well as to enhance their reproducibility^66–69^. All analyses were performed at least in triplicate.

Typically, a 10 mg/mL solution of a given dry plant extract in methanol was used. In cases where the absorbance of any of the samples was higher than 1 A.U. (or lower than 0.15 A.U. in the case of ABTS assay), the determinations were repeated using a tenfold diluted plant extract solution (i.e. 1 mg/mL, 0.1 mg/mL).

Blanks for each determination were performed in parallel to the samples, using pure methanol instead of the plant extract. For colored samples, the background absorbance was measured in a similar manner, replacing the appropriate reagent with water.

Additionally, in the case of calendula and chamomile extracts, due to the high content of lipophilic compounds, tetrahydrofuran (THF) was used as a solvent instead of methanol. Non-activity of THF in all of the assays was checked beforehand.

All determinations were performed on a Jenway 7415 spectrophotometer (Cole-Parmer, UK).

#### *Determination of *in vitro* anti-radical properties (ABTS method)*

The assay reagent was prepared by mixing 1.5 mL of pre-prepared ABTS solution (14 mM in H_2_O, i.e., 38.4 mg in 5 mL) and 1.5 mL of potassium persulfate solution (7 mM in H_2_O, i.e., 33.8 mg in 25 mL) in a closed, screw-cap vial and left in the dark at room temperature for 14–20 h. At the end of this time, the assay reagent was diluted to a volume of 200 mL. The absorbance of solution should be in the range of 0.775 ± 0.025 AU. The determinations were made by mixing 3 mL of the reagent and 100 μL of methanolic solutions of dry plant extracts. After that, the mixture was left in the dark for exactly 6 min, followed by an absorbance measurement at 734 nm. The obtained absorbance values ​​should fit in the range of 0.15—0.70 AU. The free radical scavenging ability of the sample is calculated from the following formula:

$$\% I\, = \,[(A_{0} {-}A_{s} )/A_{0} ]*{1}00.$$

Where: *%I*—inhibition percentage, *A*_*0*_—blank sample absorbance, *A*_*s*_—sample absorbance.

A standard curve was prepared by replacing the extract solution with a methanolic solution of Trolox in variable concentration (0.15, 0.12, 0.09, 0.06, 0.03 mg/mL).

#### Ferric ion reducing antioxidant parameter (FRAP)

To prepare the reagent, a 300 mM acetate buffer (3.0 g of CH_3_COONa and 4.1 mL of conc. CH_3_COOH for 250 mL), a 10 mM solution of tripyridyltriazine (TPTZ) in HCl (75 mg of TPTZ and 86 uL of 36% HCl for 25 mL), and a 20 mM solution of FeCl_3_·6H_2_O (135 mg for 25 mL) were prepared. On the day of analysis, the acetate buffer, TPTZ and FeCl_3_ solutions were combined in a 10:1:1 (v/v/v) ratio to obtain the FRAP reagent, which was placed in a heating bath (37 °C) for about 10–15 min prior to use. For the determination, 3.2 mL of warm reagent, 200 μL of methanol and 100 μL of methanolic plant extract solution were mixed and left for 4 min in a closed screw-cap vial. After this time, the absorbance of the samples was measured at 593 nm (the color is more stable than in the case of ABTS assay).

Different volumes (20, 40, 60, 80, 100, 120, 140 μL) of an aqueous solution of FeSO_4_·7H_2_O (1 mM, 27.2 mg in 100 mL H_2_O) and methanol (adding up to the total volume of 300 μL added to 3.2 mL of FRAP reagent) were used to prepare the standard curve.

#### Determination of total phenolic compounds (Folin–Ciocalteau method, FC)

Determinations were performed by mixing 2.5 mL of a tenfold diluted Folin–Ciocalteau's standard reagent and 100 µL of a methanolic plant extract solution in a 4 mL screw-cap vial. After 30 s, 0.5 mL of 20% aqueous Na_2_CO_3_ was added to the solution, and it was left for 2 h in the dark, after which the absorbance was measured at 760 nm. The standard curve was prepared using gallic acid (0.20, 0.16, 0.12, 0.08, 0.04, 0.02 mg/mL in methanol).

#### Flavonoid content determination (complexometric method with Al^3+^ ions, FL-Al)

The reagent used for determinations was an aqueous solution of Al(NO_3_)_3_·9H_2_O (57 mg/mL). Samples for measurements were prepared by mixing 900 µL of methanol, 100 µL of methanolic solutions of plant extracts and 1 mL of reagent solution, shaken and left for 5 min. Absorbance measurements were performed at 420 nm (the color is stable for 1–2 h; care should be taken that the absorbance fits in the range of 0.1–1.0 AU). Additionally, it should be noted that many plant extracts have an inherent, yellowish color, making background absorption measurements obligatory for this assay (despite the high dilutions used).

Quercetin was used as a standard (solutions with concentrations in the range of 0.015–0.150 mg/mL). The measurements were carried out in the same way as in the case of extract samples, except for replacing their methanolic solutions with different standard solutions.

### Fatty acid analysis

Dry extract samples weighing 9–15 mg were used for the analysis. To the material samples, 0.5 mL of *t*-butyl methyl ether, 0.25 mL of trimethylsulfonium hydroxide solution (0.25 M in methanol) and 25 μL of internal standard (methyl undecanoate; 105 mg in 10 mL of *t*-butyl methyl ether) were added. The analyses were performed using a Varian 450-GC gas chromatograph (Agilent Technologies, USA).

The identification of fatty acid methyl esters (FAME) was performed by comparing the retention times of samples with the retention times of the standards. The fatty acid content was calculated as percentage of sample mass (detailed information is available in the Supplementary material).

### Elemental analysis (ICP-OES)

The analysis of important micro- and macroelements (potassium, calcium, magnesium, sodium, chromium, iron, zinc, copper, silver, cobalt) in dry plant extracts was performed using inductively coupled plasma optical emission spectrometry (Quantima Sequential apparatus, GBC, Australia), after prior mineralization of samples using a mixture of hydrochloric and nitric acids (microwave mineralizer Magnum II, ERTEC, Poland).

The calibration curve was prepared by measuring the emission of standard solutions of elements in the following concentrations: 1 mg/L, 2 mg/L and 5 mg/L, and the blank sample.

Details of the analysis can be found in the Supplementary material.

## Supplementary Information

Supplementary Information.

## Abbreviations

RD: Dried damask rose petals
MC: Dried chamomile flower heads
CO: Dried calendula flowers
SN: Dried elderberry flowers
SO: Dried and cut whole sage herb
SOX: Soxhlet extraction
UAE: Ultrasound-assisted solvent extraction
MAE: Microwave-assisted solvent extraction
SFE: Supercritical carbon dioxide extraction
RD-SOX: Extract obtained from dried damask rose petals, using Soxhlet apparatus extraction
ABTS: Radical scavenging assay, based on the reaction with 2,2'-azino-bis(3-ethylbenzothiazoline-6-sulfonic acid) diammonium salt
FRAP: Ferric ion reducing antioxidant parameter, based on reaction with 2,4,6-tris(2-pyridyl)-*s*-triazine
FC: Folin–Ciocalteau assay, total phenolic content determining colorimetric method
FL-Al: Complexometric method with Al^3^^+^ ions, selective for flavonoids, and specificially flavonols
