# Supplementary material for: Phenol-rich alternatives for Rosa x damascena Mill. Efficient phytochemical profiling using different extraction methods and colorimetric assays
Source: Sci Rep. 2021 Dec 13;11:23883. doi: 10.1038/s41598-021-03337-1 (PMC8668888; doi:10.1038/s41598-021-03337-1)
Supplement: Supplementary file 1 — Supplementary Information. [file 41598_2021_3337_MOESM1_ESM.pdf]

Supplementary material

Phenol-rich alternatives for *Rosa x damascena* Mill.

Efficient phytochemical profiling using different extraction methods and colorimetric assays.

Zuzanna Piotrowicz[a], Łukasz Tabisz[a]*, Marta Waligórska[a], Radosław Pankiewicz[a], Bogusława Łęska[a]

[a] Faculty of Chemistry, Adam Mickiewicz University in Poznań, Uniwersytetu Poznańskiego 8, 61-614 Poznań

* Corresponding author: lukasz.tabisz@amu.edu.pl

Table S1. Extraction yields and antioxidant assay activities of extracts from five plant obtained using four distinct methodologies.

Raw material	Extraction				ABTS (*in vitro* radical scavenging potential)				FRAP (Ferric ion reduction potential)				Folin-Ciocalteau (total phenolics)				Al^{3+} Complexation (flavonoids)			
	Method	Material mass [g]	Extract mass [g]	Yield [%]	Extract [mgTE/g]	Material [mgTE/g]	Crop min [kgTE/ha]	Crop max [kgTE/ha]	Extract [mgFeE/g]	Material [mgFeE/g]	Crop min [kgFeE/ha]	Crop max [kgFeE/ha]	Extract [mgGAE/g]	Material [mgGAE/g]	Crop min [kgGAE/ha]	Crop max [kgGAE/ha]	Extract [mgQE/g]	Material [mgQE/g]	Crop min [kgQE/ha]	Crop max [kgQE/ha]
Calendula officinalis	SOX	10.027	3.529	35.195	20.97	7.38	3.69	10.11	9.12	3.21	1.61	4.40	45.32	15.95	7.98	21.85	19.77	6.96	3.48	9.53
	UAE	10.026	1.661	16.567	14.86	2.46	1.23	3.37	7.08	1.17	0.59	1.61	29.08	4.82	2.41	6.60	12.83	2.12	1.06	2.91
	MAE	1.009	0.210	20.813	14.59	3.04	1.52	4.16	6.95	1.45	0.72	1.98	47.29	9.84	4.92	13.48	23.65	4.92	2.46	6.74
	SFE	1.200	0.054	4.500	111.75	5.03	2.51	6.89	11.74	0.53	0.26	0.72	22.36	1.01	0.50	1.38	7.54	0.34	0.17	0.46
Matricaria chamomilla	SOX	10.004	4.132	41.303	49.66	20.51	6.15	10.26	28.56	11.80	3.54	5.90	61.56	25.43	7.63	12.71	14.64	6.05	1.81	3.02
	UAE	10.006	1.441	14.401	36.03	5.19	1.56	2.59	15.98	2.30	0.69	1.15	49.18	7.08	2.12	3.54	14.03	2.02	0.61	1.01
	MAE	1.026	0.178	17.349	42.43	7.36	2.21	3.68	21.58	3.74	1.12	1.87	52.95	9.19	2.76	4.59	16.12	2.80	0.84	1.40
	SFE	1.959	0.043	2.195	27.11	0.60	0.18	0.30	19.85	0.44	0.13	0.22	25.94	0.57	0.17	0.28	7.33	0.16	0.05	0.08
Sambucus nigra	SOX	19.754	7.406	37.491	81.72	30.64	3.12	6.46	41.92	15.72	1.60	3.32	115.92	43.46	4.43	9.17	16.23	6.08	0.62	1.28
	UAE	19.721	2.498	12.667	75.58	9.57	0.98	2.02	42.88	5.43	0.55	1.15	100.43	12.72	1.30	2.68	27.59	3.49	0.36	0.74
	MAE	2.079	0.256	12.314	82.39	10.15	1.03	2.14	45.11	5.56	0.57	1.17	102.10	12.57	1.28	2.65	20.08	2.47	0.25	0.52
	SFE	4.085	0.096	2.350	11.71	0.28	0.03	0.06	6.40	0.15	0.02	0.03	23.25	0.55	0.06	0.12	15.43	0.36	0.04	0.08
Salvia officinalis	SOX	19.368	2.442	12.608	101.91	12.85	5.76	21.60	56.51	7.13	3.19	11.98	74.79	9.43	4.22	15.85	18.28	2.31	1.03	3.87
	UAE	19.330	1.353	6.999	81.72	5.72	2.56	9.62	37.82	2.65	1.19	4.45	61.78	4.32	1.94	7.27	12.93	0.90	0.41	1.52
	MAE	2.081	0.180	8.650	87.69	7.59	3.40	12.75	44.98	3.89	1.74	6.54	65.38	5.65	2.53	9.51	17.26	1.49	0.67	2.51
	SFE	3.252	0.155	4.766	135.65	6.47	2.90	10.87	55.83	2.66	1.19	4.47	80.87	3.85	1.73	6.48	10.70	0.51	0.23	0.86
Rosa damascena	SOX	10.006	4.423	44.203	708.46	313.17	20.67	50.42	203.54	89.97	5.94	14.49	233.93	103.41	6.82	16.65	20.49	9.06	0.60	1.46
	UAE	10.056	2.536	25.219	177.57	44.78	2.96	7.21	69.74	17.59	1.16	2.83	106.15	26.77	1.77	4.31	16.61	4.19	0.28	0.67
	MAE	1.042	0.279	26.775	291.44	78.04	5.15	12.56	93.91	25.14	1.66	4.05	64.16	17.18	1.13	2.77	20.75	5.56	0.37	0.89
	SFE	1.345	0.134	9.963	18.14	1.81	0.12	0.29	2.34	0.23	0.02	0.04	8.03	0.80	0.05	0.13	7.14	0.71	0.05	0.11

Table S2. Fatty acid and microelement content from five plant obtained using four distinct methodologies.

Raw material	GC - Saturated fatty acids				GC - Unsaturated fatty acids				ICP-OES [mg microelement/g of extract]					
	Extract [mgSFA/g]	Material [mgSFA/g]	Crop min [kgSFA/ha]	Crop max [kgSFA/ha]	Extract [mgUFA/g]	Material [mgUFA/g]	Crop min [kgUFA/ha]	Crop max [kgUFA/ha]	Na	Zn	Fe	Cu	Cr	Total
Calendula officinalis	40.00	14.08	7.04	19.29	21.00	7.39	3.70	10.13	0.050	0.000	0.024	0.000	0.000	0.075
	29.00	4.80	2.40	6.58	16.00	2.65	1.33	3.63	0.033	0.000	0.017	0.000	0.000	0.050
	61.00	12.70	6.35	17.39	21.00	4.37	2.19	5.99	0.033	0.000	0.015	0.000	0.000	0.049
	75.00	3.38	1.69	4.62	26.00	1.17	0.59	1.60	0.004	0.000	0.002	0.000	0.000	0.006
Matricaria chamomilla	25.00	10.33	3.10	5.16	42.00	17.35	5.20	8.67	0.030	0.001	0.010	0.000	0.000	0.041
	26.00	3.74	1.12	1.87	44.00	6.34	1.90	3.17	0.017	0.000	0.008	0.000	0.000	0.025
	31.00	5.38	1.61	2.69	45.00	7.81	2.34	3.90	0.015	0.000	0.008	0.000	0.000	0.023
	53.00	1.16	0.35	0.58	55.00	1.21	0.36	0.60	0.002	0.000	0.001	0.000	0.000	0.003
Sambucus nigra	14.00	5.25	0.54	1.11	24.00	9.00	0.92	1.90	0.000	0.000	0.005	0.000	0.001	0.007
	30.00	3.80	0.39	0.80	51.00	6.46	0.66	1.36	0.000	0.000	0.003	0.000	0.000	0.004
	47.00	5.79	0.59	1.22	79.00	9.73	0.99	2.05	0.000	0.000	0.003	0.000	0.000	0.003
	54.00	1.27	0.13	0.27	112.00	2.63	0.27	0.56	0.000	0.000	0.000	0.000	0.000	0.000
Salvia officinalis	18.00	2.27	1.02	3.82	25.00	3.15	1.41	5.30	0.001	0.000	0.010	0.000	0.000	0.011
	25.00	1.75	0.78	2.94	30.00	2.10	0.94	3.53	0.000	0.000	0.007	0.000	0.000	0.007
	22.00	1.90	0.85	3.20	25.00	2.16	0.97	3.64	0.000	0.000	0.008	0.000	0.000	0.008
	24.00	1.14	0.51	1.92	30.00	1.43	0.64	2.40	0.000	0.000	0.001	0.000	0.000	0.001
Rosa damascena	3.00	1.33	0.09	0.21	2.00	0.88	0.06	0.14	0.000	0.001	0.053	0.001	0.014	0.069
	7.00	1.77	0.12	0.28	5.00	1.26	0.08	0.20	0.000	0.000	0.054	0.000	0.013	0.067
	9.00	2.41	0.16	0.39	5.00	1.34	0.09	0.22	0.000	0.000	0.054	0.000	0.014	0.068
	29.00	2.89	0.19	0.47	17.00	1.69	0.11	0.27	0.000	0.000	0.003	0.000	0.002	0.004

Table S3. Detailed fatty acid composition of plant extracts obtained by four different methodologies. Fatty acids are shown as total **carbon atoms : unsaturated bonds**. Results are given as % of extract mass (**+** denotes concentration above LOD, but below LOQ).

Raw material	Extraction	6:0	8:0	10:0	12:0	14:0	15:0	16:0	16:1	17:0	18:00	18:1	18:2	18:3	20:0	20:1	20:2	20:3 n-6	20:3 n-3	20:4	21:0	22:0	22:1	22:2	23:0	24:0	24:1
Calendula officinalis	SOX			+	0.2	1.1		2.1	0.1	0.1	0.3	0.1	1.0	0.8	0.1		0.1		+			0.1					
	UAE		+	+	0.1	0.7		1.6	+	0.1	0.3	0.1	0.8	0.6	+		0.1					0.1		+			
	MAE		0.1	0.1	0.4	1.8		2.8	+	0.1	0.6	0.3	1.0	0.7	+		0.1		+		+	0.1			+	0.1	
	SFE		0.1	0.0	0.4	2.4	+	3.7	0.1	0.1	0.7	0.3	1.1	1.0	0.1				0.1								
Matricaria chamomilla	SOX			0.3	+	0.1	+	1.7	0.1	0.1	0.2	0.5	2.3	1.3	0.1		+		+					+			
	UAE	+	+	0.2	+	0.1	+	1.7	0.1	0.2	0.2	0.4	2.4	1.5	0.1				+	+		0.1					
	MAE	+	+	0.3	+	0.1	+	1.9	+	0.1	0.3	0.5	2.5	1.5	0.1				+	+	+	0.1				0.2	+
	SFE	0.1	0.1	0.6	0.1	0.2	+	2.5	+	0.1	0.5	0.7	2.9	1.6	0.2				0.1	0.2	+	0.3	+			0.6	
Sambucus nigra	SOX			+		+		1.2	0.1	+	0.1	0.4	1.0	0.9	+		+		+			+		+		0.1	
	UAE		+	+		+	+	2.2	0.1	0.2	0.2	1.0	2.0	2.0	0.1		+		+		+	0.1	+	+		0.2	+
	MAE	+	+	0.1	+	0.1	+	3.6	0.2		0.3	1.6	3.0	3.0	0.1	0.1	+	+		+	+	0.2	+	+		0.3	+
	SFE			+	+	0.2	+	4.5	0.3	0.1	0.4	3.0	3.8	4.0	0.2		+		+				0.1	+			
Salvia officinalis	SOX			+	+	+		1.2	0.1	0.1	0.2	0.6	0.7	1.0	0.1	+	0.1			+		0.1	+			0.1	
	UAE		+	0.1	+	+		1.6	+	0.1	0.3	0.7	0.9	1.3	0.1	+	0.1			+	+	0.1	+			0.2	
	MAE		+	0.1	0.1	+		1.5	+	0.1	0.3	0.6	0.8	1.0	0.1		0.1		+								
	SFE				+	+		1.4	0.1	0.1	0.3	0.7	0.9	0.8	0.1	+	0.1	0.2	0.2			0.1				0.4	
Rosa damascena	SOX		+	+	+	+		0.2		+	0.1	0.1	+	0.1	+							+					
	UAE		0.0	0.1	0.0	0.0		0.4	+	+	0.2	+	0.1	0.4	+							+				+	
	MAE		0.1	0.0	0.1	0.1		0.4		+	0.2	0.1	0.1	0.3	+							+				+	
	SFE		0.0	0.1	0.2	0.1		1.2	+	0.1	0.6	0.1	0.8	0.7	0.2							0.2		0.1		0.2	

Table S4. Detailed microelement composition of extracts obtained by four different methodologies.

Raw material	Extraction	Amount of microelement in extract [ppm]					
		Na	Zn	Fe	Cu	Cr	Σ
Calendula officinalis	SOX	50.23	0.248	23.89	0.193	<LOQ	74.561
	UAE	33.22	0.021	16.82	0.138	<LOQ	50.199
	MAE	33.21	0.021	15.44	0.128	<LOQ	48.799
	SFE	4.052	0.016	1.976	0.007	<LOQ	6.051
Matricaria chamomilla	SOX	30.01	0.52	10.11	0.221	<LOQ	40.861
	UAE	16.92	0.057	8.021	0.131	<LOQ	25.129
	MAE	15.23	0.067	7.942	0.134	<LOQ	23.373
	SFE	1.823	0.032	1.023	0.015	<LOQ	2.893
Sambucus nigra	SOX	0.443	0.322	5.021	0.154	0.722	6.662
	UAE	0.289	0.178	3.021	0.081	<LOQ	3.569
	MAE	0.227	0.167	2.986	0.078	<LOQ	3.458
	SFE	0.034	0.012	0.097	0.003	<LOQ	0.146
Salvia officinalis	SOX	0.5	0.11	10.112	0.081	<LOQ	10.803
	UAE	0.38	0.09	6.901	0.042	<LOQ	7.413
	MAE	0.337	0.087	7.772	0.043	<LOQ	8.239
	SFE	0.276	0.051	0.543	0.02	<LOQ	0.890
Rosa damascena	SOX	0.423	0.671	52.61	1.121	14.25	69.075
	UAE	0.231	0.294	53.65	0.134	12.71	67.019
	MAE	0.233	0.325	53.62	0.134	13.52	67.832
	SFE	0.112	0.021	2.601	0.032	1.501	4.267
